# Association of postmigration stressors and intolerance of uncertainty to posttraumatic stress disorder in asylum seekers: the moderating role of environmental sensitivity

**DOI:** 10.3389/fpsyg.2025.1441946

**Published:** 2025-07-30

**Authors:** Ughetta Moscardino, Chiara Ceccon, Libera Ylenia Mastromatteo, Sara Scrimin, Francesca Lionetti, Michael Pluess

**Affiliations:** ^1^Department of Developmental Psychology and Socialisation, University of Padova, Padua, Italy; ^2^Department of Biological and Experimental Psychology, Queen Mary University of London, London, United Kingdom; ^3^Department of Brain and Behavioral Sciences, University of Pavia, Pavia, Italy; ^4^School of Psychology, Faculty of Health and Medical Sciences, University of Surrey, Guildford, United Kingdom

**Keywords:** environmental sensitivity, sensory processing sensitivity, postmigration living difficulties, intolerance of uncertainty, posttraumatic stress disorder, asylum seekers

## Abstract

**Introduction:**

Asylum seekers are frequently exposed to severe pre- and postmigration stressors that place them at elevated risk for posttraumatic stress disorder (PTSD). While much is known about trauma exposure, less research has examined how individual differences in environmental sensitivity—defined as heightened responsiveness to contextual influences—shape mental health outcomes in this population. This study explores how postmigration living difficulties and intolerance of uncertainty relate to specific PTSD symptom clusters, and whether environmental sensitivity moderates these associations.

**Methods:**

Participants were 157 male asylum seekers (*M*_age_ = 26.3 yrs, *SD* = 6.05) mostly originating from West Africa (89%) and hosted in second-line facilities in Northeastern Italy. Asylum seekers were individually interviewed by trained researchers using questionnaires on postmigration living difficulties, intolerance of uncertainty, environmental sensitivity, and posttraumatic stress symptoms.

**Results:**

Commonly reported stressors were lack of work (83%), family separation (77%), fear of deportation (72%), and delays in asylum processing (69%). Overall, 41% of participants exceeded the clinical cutoff for PTSD. Bivariate analyses indicated that postmigration stressors and intolerance of uncertainty were both associated with greater negative cognitions/affect and hyperarousal; postmigration stressors were also related to increased intrusion. In regression models, environmental sensitivity moderated these effects: among individuals facing high postmigration stressors, those low in sensitivity reported fewer avoidance symptoms. Conversely, among individuals with high intolerance of uncertainty, those with average or high sensitivity reported more negative cognitions/affect than their less sensitive peers.

**Discussion:**

Postmigration stressors and uncertainty contribute uniquely to PTSD symptomatology among asylum seekers, with environmental sensitivity shaping how these risk factors manifest. Environmental sensitivity-informed interventions (e.g., emotion regulation support for highly sensitive individuals) and policies aimed at reducing uncertainty and structural barriers could help buffer psychological distress in this vulnerable population.

## 1 Introduction

Worldwide, there are currently 6.1 million people seeking asylum (i.e., individuals who fled their country and are seeking protection in another country but have not yet been legally recognized as refugees; UNHCR, 2023). Of these, roughly 1 million are resettled in countries of the European Union, with Italy having a prominent role in the reception of these migrants due to its geographical position. In addition to experiencing a host of pre-, peri, and posttraumatic events related to torture, war, and political violence in their countries of origin and/or during displacement (Silove et al., 2017), asylum seekers often find themselves living in a “limbo” for prolonged periods of time due to the uncertainty and uncontrollability of the international protection application process (Ceccon and Moscardino, 2022; Solberg et al., 2021). Moreover, they are frequently exposed to postmigration living difficulties (e.g., precarious living conditions, social isolation, insecurity regarding legal status) that may compromise their mental health and psychosocial functioning (Steel et al., 2009). Indeed, previous research indicates that asylum seekers have a higher prevalence of mental health problems in comparison to both the general population (Blackmore et al., 2020) and to refugees who were granted a formal stay permit or refugee status, with post-traumatic stress disorder (PTSD) being among the most frequently observed conditions in this population (Turrini et al., 2017).

In previous research, scholars have framed asylum seekers' psychological adjustment within a diathesis-stress/dual risk approach (Solberg et al., 2020; Steel et al., 2009), in which the vulnerability resulting from their precarious existential condition interacts with stressful events in the postmigration environment to explain mental health outcomes. However, not all individuals are equally susceptible to the effects of negative life circumstances. Here, we propose that one important factor explaining such variability is environmental sensitivity, a common trait defined as the ability to register, process, and respond to stimuli (Pluess, 2015). Several person-environment interaction models and related empirical findings suggest that highly sensitive individuals are more vulnerable to the negative impact of adverse circumstances, but that they also benefit more from positive experiences (i.e., differential susceptibility theory; Belsky and Pluess, 2009). However, the extent to which environmental sensitivity may moderate the effects of postmigration stressors on asylum seekers' PTSD symptomatology remains unclear, and little is known about the associations of these variables with the DSM 5-based four symptom clusters of PTSD (i.e., intrusion, avoidance, negative cognitions/affect, and hyperarousal; American Psychiatric Association, 2013).

The current study aimed to address this gap by investigating the contribution of postmigration living difficulties and intolerance of uncertainty to asylum seekers' PTSD symptom clusters, and to examine whether environmental sensitivity moderates these associations. Considering the at-risk living conditions of this underrepresented population, identifying for whom these conditions are more detrimental and in which specific domains of PTSD is paramount to provide tailored interventions and inform policies related to the management of the asylum process.

### 1.1 Postmigration living difficulties, intolerance of uncertainty, and PTSD

Upon arrival in the receiving country, asylum seekers often face a host of challenges that represent severe obstacles to the integration process. Common stressors include living conditions (e.g., precarious accommodation, unemployment, financial strain), experiences of discrimination, social isolation, acculturative stress, as well as family separation, the asylum process, and immigration policy (Li et al., 2016). Moreover, asylum seekers are more likely than the general population to have difficulties accessing adequate medical/healthcare services because of their legal status, lack of information, language barriers, and cultural differences (Satinsky et al., 2019).

While there is abundant evidence indicating that exposure to traumatic events in the country of origin is linked to higher prevalence of PTSD in refugee and asylum seeking populations, comparatively little research has focused on the effects of stressors experienced after resettlement (Hynie, 2018). A recent review found that poor social integration and a weak support system significantly contribute to increased posttraumatic stress among refugees and asylum seekers in Europe (Gleeson et al., 2020), but the impact of asylum seekers' postmigration stressors on different PTSD symptom clusters remains poorly understood. In a 2-year longitudinal study, Specker et al. (2024) found that postmigration stressors reported by a community sample of refugees resettled in Australia predicted increases in intrusion, avoidance, and negative cognitions/affect; moreover, such stressors were bidirectionally associated with hyperarousal symptoms. Thus, postmigration living difficulties play a crucial role in how displaced individuals adapt to their resettlement context and their precarious existential situation, potentially eroding their mental health in the long run.

A relevant individual factor that shapes asylum seekers' psychological adjustment after resettlement is their (in)ability to tolerate uncertain, unexpected or ambiguous situations. Intolerance of uncertainty can be defined as a tendency to experience fear and discomfort in the face of uncertain and unpredictable situations (Carleton, 2012). It is associated with intense negative emotional reactions to uncertain and ambiguous events regardless of their probability of occurrence, as well as to avoidant behavioral tendencies aiming to reduce anxiety (Osmanaǧaoǧlu et al., 2018). Given the temporary, transitional situation in which asylum seekers spend several months (or even years) awaiting a decision or resolution from the authorities as regards the outcome of their asylum application, the ability to manage the uncertainty and uncontrollability of this lengthy process likely plays a key role in their overall mental health (Ceccon and Moscardino, 2022; Nickerson et al., 2023).

Research with both clinical and community samples has shown that high levels of intolerance of uncertainty are linked to excessive worries and anxiety-related problems (Ceccon and Moscardino, 2024; Rosser, 2019), general psychopathology (Boswell et al., 2013), and PTSD symptom severity following trauma exposure (Badawi et al., 2022; Hollingsworth et al., 2018). Although intolerance of uncertainty may have a relevant impact on displaced individuals in light of the uncertain outcome of the refugee determination process, to our knowledge, only one study has investigated its association with PTSD among displaced populations. Specifically, Nickerson et al. (2023) found that functional impairments associated with intolerance of uncertainty were associated with subsequent increases in PTSD symptoms among displaced refugees living in Indonesia. However, the authors did not differentiate between the four PTSD symptom clusters. In a study of treatment-seeking veterans in the US, significant associations between intolerance of uncertainty and the PTSD symptom clusters of avoidance and hyperarousal were reported (Raines et al., 2019). Yet, the extent to which this pattern may generalize to asylum seekers and displaced people resettled in countries with different reception policies and migration patterns is still unknown. Thus, more research is warranted to better understand the role of intolerance of uncertainty in asylum seekers' posttraumatic stress to inform intervention approaches aimed at increasing coping abilities and minimizing mental distress among displaced populations living in transit.

### 1.2 The role of environmental sensitivity

Individuals' sensitivity to environmental influences has been defined as the “the ability to register, process, and respond to external factors” (Pluess, 2015, p. 138) and is crucial to adapt successfully to one's social and physical environment. Building on previous theoretical models of person-environment interactions and their role in adjustment quality (Aron et al., 2012; Belsky and Pluess, 2009; Boyce and Ellis, 2005), Pluess (2015) proposed a meta-framework integrating several theories of individual-environment interaction under the same umbrella and considers environmental sensitivity as an inherited trait responsible for individual differences in response to internal and external stimuli. This trait has been shown to increase vulnerability when people are faced with negative events, consistent with the diathesis-stress model where environmental sensitivity functions as a diathesis or predisposition factor that amplifies the impact of stress. At the same time, sensitive individuals may derive greater benefit from supportive environments, reflecting principles of vantage sensitivity and differential susceptibility (Pluess and Belsky, 2013). Therefore, people substantially differ in their levels of environmental sensitivity, with some being more affected by both negative and positive contextual conditions (Aron et al., 2012).

Meta-analytic evidence suggests that environmental sensitivity, as captured by the phenotypical trait of sensory processing sensitivity (Aron and Aron, 1997), positively correlates with common personality traits (i.e., neuroticism and openness) as well as with negative affect (i.e., depression, anxiety, stress) (Lionetti et al., 2019a), although it does not overlap with traditionally considered personality characteristics. Results from prior research also support the moderating role of environmental sensitivity in a variety of contexts. For instance, increased environmental sensitivity is linked to more adjustment problems among adults reporting poor nurturing environments and adverse childhood experiences (Booth et al., 2015; Liss et al., 2008), and highly sensitive adults are more affected by the exposure to negative media pictures (Rubaltelli et al., 2018). Conversely, individuals with high sensitivity benefit more from a positive family environment (Lionetti et al., 2019b), and highly sensitive employees are more responsive to positive working conditions to achieve their best performance and thrive in the workplace (Vieregge et al., 2023). In regards to the refugee context, Karam et al. (2019) found that the link between war exposure and PTSD was the strongest in highly sensitive Syrian children with lower levels of childhood adversities, and was less pronounced in sensitive children who experienced more childhood adversities. In a related study, a direct and positive association was found between environmental sensitivity and PTSD severity among Lebanese children with no direct exposure to war. Furthermore, for highly sensitive children, the link between childhood adversities and PTSD was stronger than for those with low and medium levels of this characteristic, supporting a diathesis-stress model (Karam et al., 2024).

In the context of asylum seekers, environmental sensitivity offers a distinct perspective on psychological vulnerability, complementing better-known constructs such as resilience or emotion regulation. Whereas the latter emphasize adaptive strategies or outcomes, environmental sensitivity reflects a more basic trait-level difference in how individuals register and react to environmental input (Pluess, 2015). This distinction is particularly relevant for asylum seekers, who often face unpredictable and overstimulating environments. Highly sensitive individuals may be more reactive to such conditions, placing them at greater risk for distress if support systems are lacking. Examining sensitivity as a moderator may thus help explain heterogeneity in PTSD symptom expression beyond group-level factors such as migration status or trauma exposure.

### 1.3 The present study

The overall goal of this cross-sectional study was to examine the role of postmigration stressors, intolerance of uncertainty, and environmental sensitivity in posttraumatic stress among young adults seeking asylum in Italy. Despite growing interest in risk and resilience factors among asylum seekers, environmental sensitivity has been largely overlooked, especially in studies involving adult populations resettled in high-income countries. By addressing this gap, the present study offers a novel contribution to the literature on individual variability in posttraumatic stress among forcibly displaced individuals.

The first aim was to assess the associations of postmigration living difficulties and intolerance of uncertainty to the four DSM 5-related PTSD symptom clusters. Based on prior research on asylum seekers' mental health and trauma exposure, we expected that higher levels of postmigration stressors and more intolerance of uncertainty would be significantly and positively related to the severity of the four PTSD symptom clusters. More specifically, it was anticipated that postmigration stressors would be associated with increased symptoms of intrusion, avoidance, and negative cognitions/affect (see Specker et al., 2024), and that intolerance of uncertainty would be associated with increased avoidance and hyperarousal (Raines et al., 2019).

The second aim was to examine whether environmental sensitivity moderated these associations, in line with the diathesis-stress model (Solberg et al., 2020). Accordingly, we hypothesized that individuals with higher levels of environmental sensitivity would show stronger associations between postmigration stressors and PTSD symptom severity, as well as between intolerance of uncertainty and PTSD symptoms, compared to those with lower sensitivity. Conversely, we expected low-sensitive individuals to show weaker or no associations between these risk factors and PTSD outcomes. Given the limited literature addressing cluster-specific effects, no a priori hypotheses were formulated for individual PTSD symptom clusters.

## 2 Method

### 2.1 Study context

The study was conducted in Italy, a country that plays a particularly relevant role in the European asylum context due to its geographic position as a principal entry point for migrants crossing the Mediterranean. In 2023, it was the first country of arrival for migrants in Europe through the Mediterranean sea. The country currently hosts around 160,000 refugees and asylum seekers (UNICEF, 2024), accounting for 12% of all first-time asylum applicants in the European Union (Eurostat, 2024). Most individuals seeking international protection originate from West Africa (e.g., Nigeria, Senegal, Gambia, Mali, Ivory Coast), to a lesser extent from Southern Asia (i.e., Pakistan and Bangladesh), and more recently from Ukraine; of these, 95% are male (European Union Agency for Asylum, 2023). Italy's asylum policy and reception system, known as “accoglienza diffusa,” promote decentralized hosting in smaller facilities throughout the country rather than large centralized camps. Hence, following their identification and submission of their request for asylum, these individuals are transferred to second-line reception facilities, which are, for the most part, emergency centers run by social cooperatives. Compared to other EU countries, Italy's system involves longer bureaucratic procedures, greater policy instability, and more variable reception conditions: at the time of data collection, the waiting time for the approval (or rejection) of the asylum application lasted an average of 3.5 years (European Commission for the Efficiency of Justice, 2018). Thus, asylum seekers experience a prolonged “limbo,” marked by great uncertainty about future developments of their legal situation and the inability to reclaim the social roles and identities previously held in the country of origin. Despite having a legal residence permit and participating in activities such as Italian language classes and professional training, during the asylum procedure these individuals still face several barriers in accessing services reserved for Italian citizens and in securing stable employment (Ceccon and Moscardino, 2022). For instance, employers often hesitate to hire individuals with pending legal status and may exploit their migrant condition by offering underpaid jobs without long-term security. Hence, throughout the whole procedure, asylum seekers need to rely heavily on reception facilities and staff, which may result in a failure to foster their autonomy and effective inclusion into the host society. Overall, these features underscore Italy's particular relevance for examining the mental health impact of postmigration stressors and systemic uncertainty.

### 2.2 Participants

Data collection was conducted over a period of approximately 3 years, from November 2016 to May 2019. Participants were recruited through informal contacts with social cooperatives managing second-line residential facilities in northeastern Italy. Inclusion criteria were (a) being 18 years or older, and (b) having a pending asylum application in Italy. We excluded individuals with a formal psychiatric diagnosis or who were currently receiving pharmacological treatment, as identified in consultation with social workers and mental health professionals affiliated with the facilities. Although the interview did not include highly sensitive clinical content, this exclusion aimed to safeguard asylum seekers in a clinically vulnerable state and to reduce potential confounding effects of ongoing psychiatric conditions on PTSD symptom reporting. Importantly, this criterion did not exclude participants experiencing psychological distress in the absence of a formal diagnosis.

A total of 191 young adult asylum seekers agreed to take part in the study and provided written informed consent. Of these, seven did not meet the inclusion criteria, two had obtained some form of international protection, 12 were unavailable after the introductory meeting, and 13 withdrew or canceled due to work or personal reasons. The final sample was composed of 157 asylum seekers. To ensure safety and comfort, participants were informed before the interview about the availability of mental health professionals (i.e., psychologists, psychotherapists, psychiatrists) affiliated with the social cooperatives or the local health districts, whom they could contact in case of need.

Participants' mean age was 26.3 years (*SD* = 6.05, range = 18–45). In line with national statistics concerning the asylum seeking population in Italy, the sample was composed of male participants mainly originating from West African countries (Nigeria, 41%; Ivory Coast, 13%; Mali, 11%; Gambia, 11%; Senegal, 7%; Ghana, 6%). All but one participant had arrived in Italy via the Mediterranean route departing from Libya, and most of the asylum seekers (89%) reported that their family members were still living in the country of origin. Participants had been residing in Italy for an average of 20 months (*SD* = 6, range = 2–44); 59% had submitted one asylum application, and the remaining had submitted two or more applications (i.e., appeal). Regarding education, 9% of participants had no education, 26% had completed primary school, 56% had completed secondary school, and 9% had a university degree. At the time of data collection, the majority was unemployed (86%), 14% were students, 13% were employed, and 9% were studying and working.

### 2.3 Procedure

Before data collection, the study protocol and procedures were approved by the Ethics Committee of the School of Psychology at the University of Padova. Written informed consent was collected from participants at the beginning of each interview. Researchers emphasized that participation was voluntary, that the study would not influence their asylum application and/or living conditions, and that no information concerning any individual would be shared with the authorities or the cooperatives' staff. Individual face-to-face interviews lasted between 70–140 min and comprised both questions from standardized questionnaires (as described below) and open-ended questions. Interviews were held at the participants' residential facilities in a quiet, separate room to ensure privacy. Interviews were conducted by trained multilingual researchers in Italian, English, or French, based on each participant's preference and the interviewers' fluency. The latter two languages were proposed in consideration of the geographical origin of most asylum seekers in this sample (i.e., anglophone or francophone African countries). Researchers participated in a 6-hour training session covering instrument familiarization, interviewing techniques, ethical considerations, and mentorship structure (i.e., less experienced interviewers were paired with more experienced colleagues during initial interviews to ensure consistency and quality control). This structured approach was designed to enhance the reliability and comparability of responses in a multilingual setting. Participants were also informed that linguistic-cultural mediators could be involved upon request to ensure accurate interpretation and culturally sensitive communication. Interviews were conducted in English (49%), Italian (29%), and French (22%). At the end of the session, asylum seekers were thanked for their participation and were offered some refreshments as well as a financial compensation (i.e., shopping voucher of €10).

### 2.4 Measures

All questionnaires included in the interview were selected based on their easiness of comprehension, prior use with refugee/immigrant populations, and brevity to minimize participants' fatigue. Unless otherwise noted, questionnaires were translated in the three respective languages (Italian, English, French) using standard translation-backtranslation methods; prior to verbal administration, they were checked by linguistic-cultural mediators for their cultural appropriateness and translation accuracy.

#### 2.4.1 Demographic characteristics

Information was collected on participants' age, country of origin, religion, educational attainment, pre-and post-migration occupation, length of residence in Italy, Italian language proficiency, family status, residence of family of origin, and number of submitted asylum applications.

#### 2.4.2 Exposure to potentially traumatic events

Participants' exposure to potential lifetime traumatic events was assessed using the DSM-5 Life Events Checklist (LEC-5; Weathers et al., 2013a). The scale includes 16 items concerning different types of events that can potentially lead to PTSD (e.g., natural disasters, accidents, physical/sexual assaults, war experiences, illnesses, injuries, or death experiences). In this study, we added three items reflecting the experience of forced migration among asylum seekers in Italy (i.e., “Separation from family,” “Forced to perpetrate violence against own family, community, nation,” “Imprisonment, detention in re-education/concentration camps and other kind of settings”). Items are rated on a scale ranging from 4 = happened to me, 3 = witnessed it, 2 = learned about it, 1 = not sure, to 0 = doesn't apply. The original version has an additional response option (“part of my job”) that was excluded in consideration of the target group. A total score was obtained by summing up the number of events reported by participants as “happened to me” in order to focus on the very strict definition of trauma exposure (see Nesterko et al., 2020). The LEC-5 is valid and reliable across cultures (Kwobah et al., 2022). In this study, internal consistency as indicated by Cronbach's alpha was 0.63.

#### 2.4.3 Postmigration stressors

The Post-Migration Living Difficulties Checklist (Silove et al., 1997; Steel et al., 1999) assesses 25 living difficulties commonly reported by refugees and asylum-seekers in the resettlement context, such as communication difficulties, discrimination, and various socioeconomic challenges. We included an additional item reflecting a common experience among asylum seekers in Italy, “hostility/rejection from people in the neighborhood.” Participants rated each living difficulty on a 5-point scale (from 0 = no serious problem to 4 = very serious problem) referring to the time since their arrival in Italy. A mean score was calculated for the present study. The scale has been found to reliably predict mental health among refugees and asylum seekers (Nickerson et al., 2010; Steel et al., 2006). In this study, internal consistency of the questionnaire was α = 0.68.

#### 2.4.4 Intolerance of uncertainty

Asylum seekers' ability to tolerate the uncertainty of ambiguous situations, cognitive and behavioral responses to uncertainty, perceived implications of uncertainty, and attempts to control the future were measured with the Intolerance of Uncertainty Scale – Short Form (Carleton et al., 2007). Participants were asked to express their level of agreement with 12 items (e.g., “I always want to know what the future has in store for me”; “When I am uncertain I can't function very well”), with response options ranging from 1 = not at all characteristic of me to 5 = entirely characteristic of me. The total score is calculated by averaging the scores of all items. The measure has been validated in various countries and with refugee populations, showing good psychometric properties (Nickerson et al., 2023). In the current study, Cronbach's Alpha was 0.76.

#### 2.4.5 Environmental sensitivity

Participants' sensitivity to environmental stimuli was assessed via the Highly Sensitive Child scale (HSC; Pluess et al., 2018), a short and adapted child version of the Highly Sensitive Person (HSP; Aron and Aron, 1997) scale. The questionnaire comprises 12 items (e.g., “I notice when small things have changed in my environment”; “I get nervous when I have to do a lot in little time”) rated on a 7-point scale ranging from 1 (strongly disagree) to 7 (strongly agree). In this study, we used the child instead of the adult version due to its brevity and to facilitate asylum seekers' comprehension of items, which are linguistically (and conceptually) more complex in the HSP scale. Indeed, various items of the adult version, even when translated into participants' preferred languages, contained abstract and/or culturally specific formulations (e.g., “I find it unpleasant to have a lot going on at once”; “I am deeply moved by the arts or music”) that were problematic for this population, which included individuals with limited educational backgrounds. The mean score was obtained by averaging all item responses. Prior validation studies indicate that this tool captures the same theoretical construct as the adult version, but in fewer items and at an accessible reading level, and it has shown good psychometric properties in terms of validity and reliability across different countries (Pluess et al., 2018; Weyn et al., 2021). After elimination of the items 7, 9 and 11 due to their negative associations with the overall construct, Cronbach's α was 0.60.

#### 2.4.6 Posttraumatic stress disorder

We used the PTSD Checklist based on the DSM-5 (PCL-5; Weathers et al., 2013a,b) to assess the presence and severity of PTSD-related symptomatology. The PCL-5 comprises 20 items covering the four symptom clusters of PTSD according to DSM-5: intrusion (5 items; e.g., “Have repeated, disturbing dreams of the stressful experience”), avoidance (2 items; e.g., “Avoid external reminders of the stressful experience”), negative cognitions/affect (7 items; e.g., “Feel distant or cut off from other people”), and hyperarousal (6 items; e.g., “Feel jumpy or easily startled”) in the past month. Response options range from 0 = not at all to 4 = extremely, and a total symptom severity score (range: 0–80) can be obtained by summing the scores for each of the 20 items. For the purpose of this study, we calculated a mean score of PTSD symptom severity for each cluster (Crombie et al., 2021). The PCL-5 is valid across multiple cultural and linguistic groups (Bockhop et al., 2022). In the current study, all symptom clusters demonstrated good internal consistency: intrusion, Cronbach's α = 0.80; avoidance, Cronbach's α = .91; negative cognitions/affect, Cronbach's α = 0.70; and hyperarousal, Cronbach's α = 0.68.

## 3 Analytic plan

All analyses were run in R, version 4.2.0 (R Core Team, 2023). After computing descriptive statistics, we verified that there were no missing data, as all responses were collected through face-to-face interviews administered by trained researchers who ensured completeness during data collection. We then conducted bivariate correlations among postmigration living difficulties, intolerance of uncertainty, environmental sensitivity, and the four PTSD cluster symptoms. Next, four linear regression models were estimated considering the four symptom clusters of PTSD as outcome variables. In these analyses, we controlled for participants' exposure to traumatic events on the basis of theoretical and empirical associations between this variable and posttraumatic symptoms (e.g., Bentley and Dolezal, 2019; Mundy et al., 2020). The distributions of the four PTSD cluster scores were not normal, but the value of skewness and kurtosis were acceptable (≥2 and < 2), and thus were considered acceptable to prove normal univariate distribution (George and Mallery, 2019).

As a first step, we tested main effects of postmigration stressors and intolerance of uncertainty on each of the PTSD symptom cluster severity scores controlling for participants' exposure to potentially traumatic events. To examine the moderating role of environmental sensitivity, all models included the main effects and two interaction terms: postmigration living difficulties × environmental sensitivity and intolerance of uncertainty × environmental sensitivity. We also controlled for participants' exposure to potentially traumatic events. These two-way interactions were specified a priori based on our diathesis-stress framework, which suggests that environmental sensitivity may modulate the psychological impact of adverse contextual factors. Our primary theoretical focus was to examine whether sensitivity moderated the effects of postmigration stressors and intolerance of uncertainty on PTSD symptom clusters. Other potential interactions (e.g., between postmigration stressors and intolerance of uncertainty) were not included, as they were outside the scope of our hypotheses and were not supported by preliminary theoretical or statistical considerations. Significant interaction effects were further examined in follow-up simple slope analyses using the *interactions* package (Long, 2019) in R. Multicollinearity was assessed by computing Variance Inflation Factors (VIFs) for all predictors in the main-effects models, all of which were below 5, indicating no multicollinearity concerns. For models including interaction terms, we used the Generalized VIF (GVIF) adjusted for degrees of freedom [GVIF∧(1/(2 × Df)], see Fox and Monette, 1992). These GVIF values also remained below 5, confirming acceptable levels of multicollinearity in the presence of interactions.

## 4 Results

### 4.1 Trauma exposure and postmigration stressors

Participants experienced an average of six lifetime potentially traumatic events; >95% of respondents reported experiencing imprisonment and detention in re-education/concentration camps. As can be seen in Table 1, the majority of respondents had experienced forced separation from family as well as physical assault and assault with a weapon. Furthermore, over half of the respondents reported having experienced captivity and severe human suffering.

**Table 1:** Exposure to potentially traumatic events (*N* = 157).

**Potentially traumatic event**	***n***	**%**
Imprisonment, detention in re-education/concentration camps and other kind of settings	155	98.7
Forced separation from family	143	91.1
Physical assault	125	79.6
Assault with a weapon	103	65.6
Captivity	97	61.8
Severe human suffering	85	54.1
Combat or exposure to a war-zone	64	40.8
Transportation accident	55	35
Life-threatening illness or injury	47	29.9
Serious accident at work, home, or during recreational activity	46	29.3
Fire or explosion	30	19.1
Natural disaster	29	18.5
Serious injury, harm, or death you caused to someone else	17	10.8
Forced to perpetrate violence against own family, community, nation	17	10.8
Exposure to toxic substance	14	8.9
Sexual assault	9	5.7
Sudden violent death	6	3.8
Other unwanted or uncomfortable sexual experience	5	3.2
Sudden accidental death	3	1.9

Postmigration stressors are reported in Table 2. The most frequently experienced stressor was being unable to find work. More than two thirds of the asylum seekers reported experiencing difficulties with separation from family, fear of being sent home, and worries about the family in the country of origin. More than half of the respondents had experienced delays in processing of their asylum application, loneliness/boredom, isolation, and communication difficulties. In addition, almost one quarter of the respondents reported experiencing hostility/rejection from people in the neighborhood.

**Table 2:** Postmigration living difficulties reported as moderately serious, serious, or very serious problem (*N* = 157).

**Postmigration living difficulty**	***n***	**(%)**
Being unable to find work	131	83.4
Separation from family	121	77.1
Fears of being sent home	113	72
Delays in processing the asylum application	108	68.8
Worries about family back home	106	67.5
Loneliness and boredom	104	66.2
Communication difficulties/language difficulties	97	61.8
Isolation	97	61.8
Unable to return home to family in an emergency	95	60.5
No permission to work	68	43.3
Discrimination	62	39.5
Difficulty adjusting to the weather/climate	59	37.6
Interviews by immigration	39	24.8
Hostility/rejection from people in neighborhood	39	24.8
Poverty (not having enough money for basic needs, food etc.)	38	24.2
Little government help with welfare (unemployment benefits, financial help)	27	17.2
Bad working conditions	24	15.3
Conflict with immigration officials	23	14.6
Worries for not getting treatment for health problems	17	10.8
Poor access to traditional foods	13	8.3
Being unable to practice own religion	13	8.3
Poor access to long-term medical care	11	7
Poor access to emergency medical care	9	5.7
Little help with welfare from charities (social services, red cross)	8	5.1
Poor access to dental care	6	3.8
Poor access to counseling services	3	1.9

### 4.2 Descriptive and preliminary analyses

The mean sample score on the PCL-5 measure was 31.33 (range 0–80). This indicates relatively severe posttraumatic symptomatology, given that a score of 31 to 33 on the PCL-5 measure has been identified as the best cut-off for a likely diagnosis of PTSD (Bovin et al., 2016; Weathers et al., 2013a,b). Statistics and correlations among the variables are shown in Table 3.

**Table 3:** Descriptive statistics and bivariate correlations among study variables (*N* = 157).

**Variable**	**Mean (SD)**	**Range**	**1**	**2**	**3**	**4**	**5**	**6**	**7**	**8**
1. Potentially traumatic events	6.80 (2.70)	1–14		0.21^**^	0.03	−0.06	0.39^***^	0.03	0.43^***^	0.37^***^
2. Postmigration living difficulties	1.30 (0.41)	0.35–2.68			0.17^*^	0.01	0.17^*^	−0.07	0.23^**^	0.36^***^
3. Intolerance of uncertainty	3.02 (0.61)	1.75–4.67				0.18^*^	0.03	−0.11	0.22^**^	0.16^*^
4. Environmental sensitivity	4.17 (0.84)	2.33–6.89					0.02	0.11	0.09	0.10
5. PTSD – Intrusion	10.04 (5.19)	0–20						0.04	0.47^***^	0.53^***^
6. PTSD – Avoidance	4.73 (2.61)	0–8							0.18^*^	−0.02
7. PTSD – Negative cognitions/affect	9.64 (5.43)	0–23								0.57^***^
8. PTSD – Hyperarousal	6.92 (4.71)	0–20								

^*^p < 0.05; ^**^p < 0.01; ^***^p < 0.001.

Potentially traumatic events were significantly and positively related to postmigration living difficulties as well as to the PCL-5 intrusion, negative cognitions/affect, and hyperarousal cluster symptoms. Postmigration living difficulties were positively associated with intrusion, negative cognitions/affect, and hyperarousal. Intolerance of uncertainty was significantly and positively related to negative cognitions/affect and hyperarousal. Finally, sensitivity to environmental influences was not associated with any of the four PTSD symptom clusters.

### 4.3 Multivariate regression models

To study the moderating role of environmental sensitivity we estimated four linear regression models, one for each PTSD symptom cluster (i.e., intrusion, avoidance, negative cognitions/affect, hyperarousal). Specifically, we tested the first-order effects of postmigration stressors, environmental sensitivity, and intolerance of uncertainty controlling for participants' exposure to potentially traumatic events, together with the two-way interactions among postmigration living difficulties and environmental sensitivity, and intolerance of uncertainty and environmental sensitivity.

As shown in Table 4, we found a significant two-way interaction between postmigration living difficulties and environmental sensitivity for the avoidance symptom cluster (*b* = 1.46, *SE* = 0.65, *CI* = [0.12; 2.70], *p* = 0.026), and a significant two-way interaction between intolerance of uncertainty and environmental sensitivity for the negative cognitions/affect symptom cluster (*b* = 1.59, *SE* = 0.77, *CI* = [0.04; 3.11], *p* = 0.042). R squared, as a measure of effect size, ranged from 0.04 (small effect on avoidance), to 0.14 (medium effect on intrusion), to 0.22 and 0.23 (medium-to-large effect size for hyperarousal and negative cognitions/affect, respectively).

**Table 4:** Multiple regression predicting PTSD symptom cluster severity scores in asylum seekers (*N* = 157).

**Predictor**	**Intrusion**		**Avoidance**		**Negative Cognitions/Affect**		**Hyperarousal**	
	***b*(*se*)**	**95% CI**	***b*(*se*)**	**95% CI**	***b*(*se*)**	**95% CI**	***b*(*se*)**	**95% CI**
Intercept	2.78 (2.82)	[−2.74; 8.30]	4.73 (1.52)^**^	[1.75; 7.71]^*^	−4.67 (2.81)	[−10.17; 0.83]	−5.29 (2.43)^*^	[−10.05; −0.54]
Exposure to potentially traumatic events	0.72 (0.15)^***^	[0.43; 1.00]	0.05 (0.08)	[−0.10; 0.20]	0.81 (0.15)^***^	[0.52; 1.09]	0.56 (0.13)^***^	[0.31; 0.80]^*^
Postmigration living difficulties	1.13 (0.96)	[−0.75; 3.02]	−0.38 (0.52)	[−1.40; 0.64]	1.49 (0.96)	[−0.40; 3.37]	3.21 (0.83)^***^	[1.58; 4.84]
Environmental sensitivity	0.23 (0.47)	[−0.69; 1.15]	0.44 (0.25)	[−0.06; 0.93]	0.55 (0.47)	[−0.37; 1.46]	0.56 (0.40)	[−0.23; 1.35]
Intolerance of uncertainty	−0.02 (0.65)	[−1.30; 1.26]	−0.55 (0.35)	[−1.24; 0.14]	1.52 (0.65)^*^	[0.25; 2.79]	0.64 (0.56)	[−0.46; 1.74]
Postmigration living difficulties X Environmental sensitivity	1.44 (1.23)	[−0.99; 3.87]	1.46 (0.65)^*^	[0.17; 2.75]	0.58 (1.21)	[−1.82; 2.98]	1.26 (1.06)	[−0.83; 3.36]
Intolerance of uncertainty X Environmental sensitivity	0.70 (0.78)	[−0.84; 2.24]	0.68 (0.41)	[−0.14; 1.50]	1.59 (0.77)^*^	[0.06; 3.11]	0.25 (0.67)	[−1.08; 1.58]
Adjusted *R*^2^	0.14		0.04		0.23		0.22	

The coefficients provided reflect the main effects prior to the inclusion of the interaction terms; ^***^p < 0.001; ^**^p < 0.01; ^*^p < 0.05; CI, Confidence Interval.

**Figure 1:**
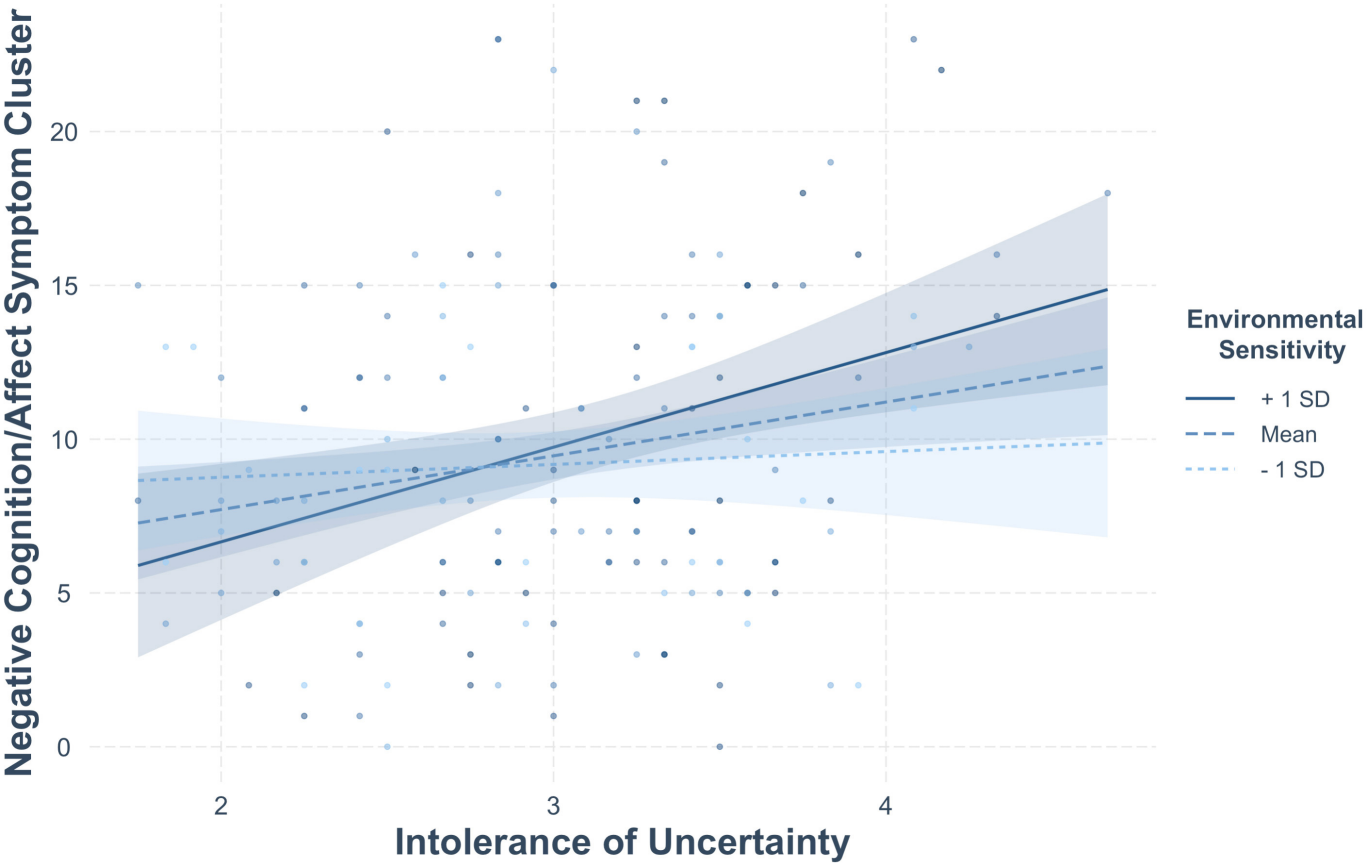
Moderating effect of environmental sensitivity on the association between intolerance of uncertainty and the negative cognitions/affect symptom cluster.

**Figure 2:**
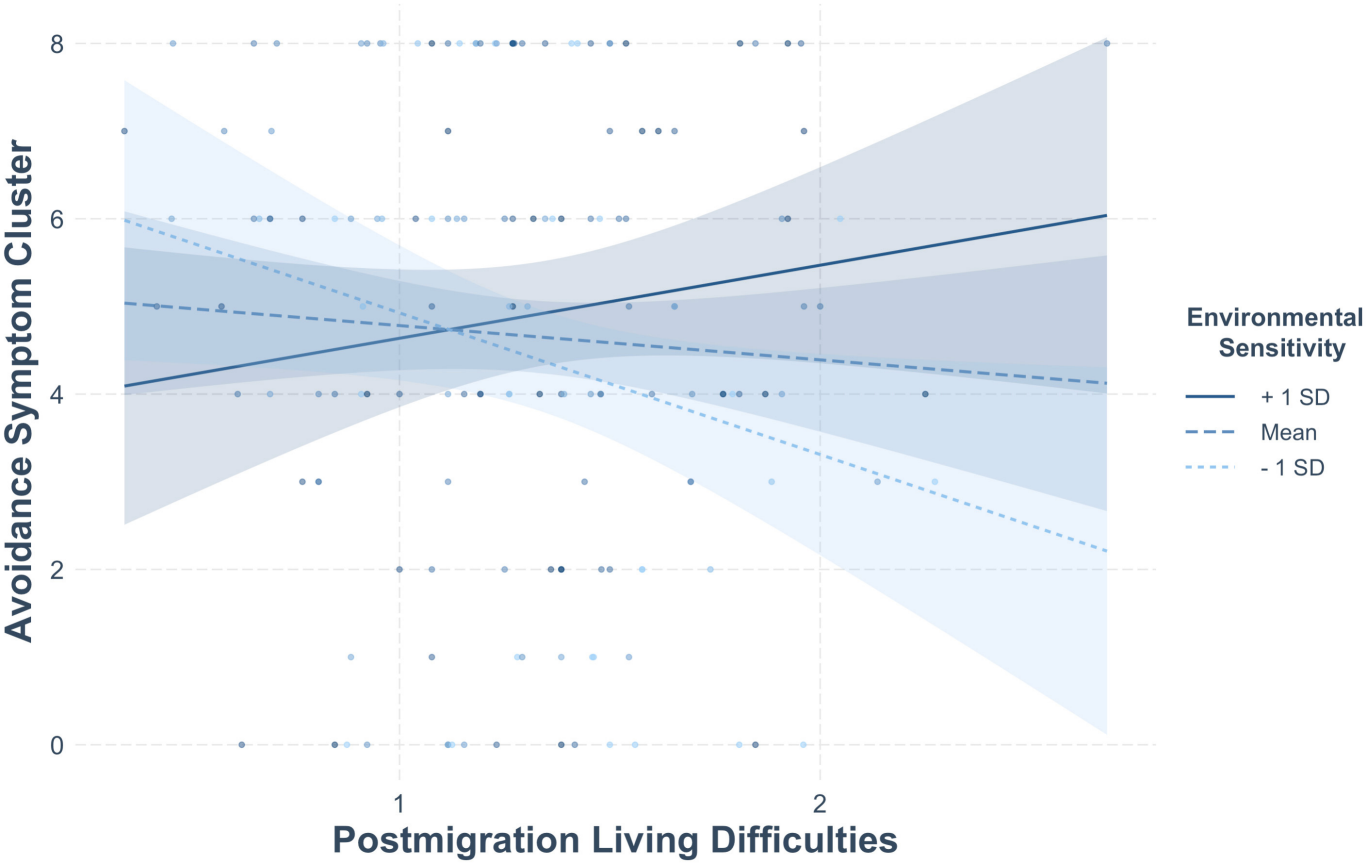
Moderating effect of environmental sensitivity on the association between postmigration living difficulties and the avoidance symptom cluster.

We conducted a simple slope analysis (Bauer and Curran, 2005) to explore significant interactions (see Figures 1, 2). Regarding avoidance, *post-hoc* simple slopes analysis revealed a statistically significant slope for postmigration living difficulties at lower levels (−1 *SD*) of environmental sensitivity (*b* = −1.61, *SE* = 0.76, *p* = 0.034), but not at medium (*b* = −0.39, *SE* = 0.51, *p* = 0.44) or higher levels of this variable (*b* = 0.83, *SE* = 0.74, *p* = 0.26). Specifically, low sensitive individuals with high levels of postmigration stressors reported less avoidance symptoms than those with average or high levels of environmental sensitivity. To further probe the interaction effect and to identify the threshold of significance for the slope of post migration living difficulties, we performed the Johnson-Neyman test, which indicated that the negative slope of post migration living difficulties was significant when environmental sensitivity values were within the [2.33; 3.54] interval. For negative cognitions/affect, simple slope analysis indicated a statistically significant slope for intolerance of uncertainty at average (*b* = 1.75, *SE* = 0.65, *p* = 0.008) and higher (+1 *SD*) levels of environmental sensitivity (*b* = 3.08, *SE* = 0.99, *p* = 0.002), but not at lower levels of this variable (−1 *SD, p* = 0.62). Hence, participants with average to high levels of environmental sensitivity reported more symptoms of negative cognitions/affect when their intolerance of uncertainty was high. This interaction effect was further explored using a Johnson-Neyman test, which indicated that the positive slope of intolerance to uncertainty was significant when environmental sensitivity values were within the [3.89; 6.89] interval.

## 5 Discussion

Asylum seekers are a vulnerable population at increased risk of developing PTSD due to the challenging life circumstances they experience before, during, and after fleeing from their home country. However, little is known about how exposure to stressors in the receiving society and individual characteristics may differentially contribute to the four DSM-5 PTSD symptom clusters, and whether a personality trait such as sensitivity to environmental influences can influence these relations. The current study aimed to fill this gap by investigating the association of postmigration living difficulties and intolerance of uncertainty to the intrusion, avoidance, negative cognitions/affect, and hyperarousal cluster symptoms, postulating moderation by environmental sensitivity.

At the bivariate level, results indicated that postmigration stressors were positively related to three of the four symptom clusters (i.e., intrusion, negative cognitions/affect, and hyperarousal), whereas intolerance of uncertainty was positively associated with negative cognitions/affect and hyperarousal. In moderation analysis, low environmental sensitivity reduced the negative impact of postmigration living difficulties on avoidance, whereas average and high environmental sensitivity exacerbated the effect of intolerance of uncertainty on negative cognitions/affect.

As regards our first aim, the positive associations between postmigration stressors and intrusion and negative cognitions/affect are consistent with prior research suggesting that contextual conditions in the receiving country (e.g., economic hardship, insecure legal status) may be detrimental for asylum seekers' mental health, especially in relation to the development of PTSD symptoms (Gleeson et al., 2020; Li et al., 2016; Nowak et al., 2023). Our findings also align with Specker et al. (2024), who found that postmigration stressors were linked to increases in intrusion and negative cognitions/affect among refugees, suggesting that these clusters are particularly sensitive to chronic environmental stress. This pattern can be explained in light of asylum seekers' elevated exposure to post-migration stressors, which could affect their ability to use effective emotion regulation strategies and therefore result in a more diffuse and persistent negative emotional state, as well as an increased vulnerability to experiencing intrusive and upsetting memories of previous traumatic events (Gross, 2015; Specker et al., 2024). Moreover, we found a significant correlation between postmigration stressors and hyperarousal, in line with a recent study investigating differential associations of such stressful events with PTSD symptom clusters (see Specker et al., 2024). The cumulative burden of post-migration living difficulties, weighing on an existing situation of pre- and peri-migration distress, might amplify the individual's reactivity to these stressors, thereby worsening hyperarousal symptoms like hypervigilance, concentration and sleep troubles, and increased startle response. Unlike prior findings, avoidance was not linked to asylum seekers' experience of postmigration stressors. Several explanations may account for this result. First, the temporal distance from trauma exposure and the average length of stay in Italy (~20 months) may have led to the attenuation or normalization of avoidance behaviors over time, such that individuals no longer identified these behaviors as symptoms and thus underreported them (Specker et al., 2024). This interpretation is consistent with research by O'Donnell et al. (2007), who found that symptoms are more prominent in the acute phase following trauma exposure and tend to diminish in relevance during chronic stages of PTSD unless reactivated by new or acute stressors. Second, the chronic and cumulative nature of postmigration stressors—such as legal uncertainty and marginalization—may not elicit the same avoidance patterns as more acute or discrete traumatic events, which have been more typically associated with avoidance responses (Bryant et al., 2018). Third, it is possible that avoidance symptoms become more prominent only when stress levels surpass a certain threshold. In contexts marked by prolonged but moderate stress, such as protracted asylum procedures, avoidance may remain less salient than symptoms like hyperarousal or negative affect. While our study did not test for non-linear associations, future research could explore threshold or curvilinear models (e.g., piecewise regressions) to examine whether avoidance becomes more predictive of distress only under conditions of extreme adversity (Alpert et al., 2021).

With respect to intolerance of uncertainty, consistent with our hypothesis, a significant association between this variable and the hyperarousal PTSD symptom cluster emerged. A possible explanation is that individuals with higher levels of intolerance of uncertainty experience increased arousal and reactive symptoms triggered by the fear and anxiety linked to uncertain post-migration conditions. Contrary to our expectations and previous findings (Oglesby et al., 2017; Raines et al., 2019), intolerance of uncertainty was unrelated to avoidance symptoms, while it significantly correlated with negative affect/cognitions. One possible explanation is that the chronic stress and ongoing insecurity typical of the asylum-seeking experience may elicit sustained emotional distress and hypervigilance, rather than behavioral avoidance (Hinton and Lewis-Fernández, 2011). Moreover, the samples used in prior studies—such as U.S. veterans and treatment-seeking community adults—differed substantially from ours in terms of sociodemographic characteristics (e.g., age, nationality) and trauma histories (e.g., combat-related trauma vs. imprisonment or forced displacement), which may account for differences in the pattern of associations with PTSD symptom clusters. Our sample consisted primarily of young adult West African men, and both cultural and contextual factors may have influenced the expression and reporting of PTSD symptoms. In many West African contexts, psychological distress is often communicated through somatic or behavioral manifestations rather than internal cognitive processes such as avoidance (Ventevogel et al., 2013). This may have contributed to the weaker observed association between intolerance of uncertainty and avoidance. Finally, systemic factors may also play a role. Studies from countries with more supportive asylum policies, such as Canada, have shown lower levels of postmigration distress (Rousseau, 2018), suggesting that both cultural norms and reception conditions jointly shape how distress is experienced and expressed in asylum-seeking populations.

In relation to our second aim, the findings revealed a moderating effect of environmental sensitivity in the association between postmigration stressors and avoidance. Specifically, individuals with low levels of environmental sensitivity experienced less avoidance symptoms in the presence of high levels of stressors compared to their average and highly sensitive counterparts. Hence, low sensitive asylum seekers might be significantly less impacted (as compared to average and high sensitive ones) by the frequency and severity of post-migration stressors, especially at the behavioral level (i.e., active avoidance of people, places, and thoughts that trigger memories of the traumatic event). This finding can be interpreted in light of the psychobiology of PTSD, particularly as regards the construct of anxiety sensitivity (i.e., a type of sensitivity that amplifies the perception of threat in various situations; Reiss and McNally, 1985). Individuals with high anxiety sensitivity tend to avoid places, people, or situations that could remind them of a traumatic event, which is a common symptom in PTSD. Following the same pattern, it is possible that people with low levels of environmental sensitivity are less prone to report avoidance symptoms due to the higher threshold needed to activate a physiological threat response. We also found that environmental sensitivity was a risk factor for increased negative cognitions/affect among average and high (vs. low) sensitive asylum seekers with high levels of uncertainty intolerance. This pattern may be understood in light of previous research showing that, whilst people with higher levels of environmental sensitivity do not necessarily experience more negative emotional states, they are indeed more susceptible to the quality of their environment. For instance, highly sensitive individuals are at heightened risk of developing internalizing problems and engaging in rumination in less supportive contexts (Lionetti et al., 2022; Yano and Oishi, 2024). Although further replication is needed to confirm these results, our study lends support to the diathesis-stress model, which considers environmental sensitivity a vulnerability when people are faced with negative events and unfavorable environments, such as a stressful post-migration living situation. Given that previous research has highlighted the increased efficacy of psychological interventions among highly sensitive, immigrant-origin youth (see Ceccon et al., 2023), the current study also paves the way for further studies investigating whether young adult asylum seekers benefit from more supportive experiences to test the vantage sensitivity hypothesis.

## 6 Limitations and future directions

Overall, the current study provides novel insights into how postmigration stressors and intolerance of uncertainty are differentially associated with the four PTSD symptom clusters, highlighting the moderating role of environmental sensitivity in some of these associations. However, several limitations should be considered when interpreting the results. First, the sample was composed exclusively of young adult males, reflecting the gender imbalance among asylum seekers in Italy, and primarily included individuals from West African countries. This limits the generalizability of findings across gender and cultural groups. Future research should aim for more diverse and balanced samples, including female asylum seekers and individuals from a wider range of geographic and cultural backgrounds, to explore potential gender-specific vulnerability profiles and cultural variation in how postmigration stressors and environmental sensitivity relate to PTSD symptomatology. Including non-asylum-seeking immigrants as a comparison group would also help clarify population-specific effects and improve generalizability. Second, participants were recruited through convenience sampling based on referrals from social workers at reception centers. Although this approach was necessary for feasibility reasons, it may have introduced selection bias, as individuals more connected to services might differ from less engaged peers in several ways (e.g., distress levels, access to support). Random or community-based sampling strategies are encouraged in future research to improve generalizability. Relatedly, several asylum seekers reported symptom levels above the PCL-5 clinical cutoff despite lacking a formal diagnosis or engagement with mental health services. Such cases are relatively common in reception centers in Italy, where individuals with undiagnosed or subclinical distress are frequently encountered. Whilst the inclusion of these participants may have introduced greater variability in distress levels than originally intended, it enhances the ecological validity of the study by reflecting the psychological heterogeneity typically observed in reception centers hosting asylum seekers. Third, although data collection spanned three years, participants were recruited cross-sectionally at different time points due to recruitment constraints. As a result, we were unable to examine changes in stress levels or symptom severity over time. This limits causal inference, as the directionality of effects among study variables cannot be established. Longitudinal research is needed to clarify temporal dynamics and capture within-person changes as asylum procedures unfold. Fourth, to preserve statistical power given our sample size, we included only trauma exposure as a covariate in the regression models. While other factors (e.g., social support or acculturative stress) might be relevant, adding them would have increased model complexity beyond what the data could support. Future studies with larger samples should incorporate such variables to better reflect the multifaceted nature of asylum seekers' adjustment. Fifth, reliance on researcher-administered surveys may have influenced participants' responses due to social desirability or cultural norms. Despite training, the use of multilingual researchers, and confidentiality procedures, cultural and linguistic biases cannot be fully excluded. Moreover, although we used standardized translation protocols, nuances may have been lost in translation. Sixth, the internal consistency of the environmental sensitivity scale (HSC) was lower than expected. This may reflect linguistic challenges, situational factors, or cultural differences in how environmental sensitivity is conceptualized and expressed. We used the child version due to its greater accessibility in this population and its conceptual overlap with the adult measure (Pluess et al., 2018), but subsequent work should consider culturally adapted versions or alternative instruments validated in diverse contexts. Complementary mixed methods (e.g., observations, focus groups, key informant interviews) could also help triangulate findings and improve construct validity. Finally, while this study focused on risk factors, future research could examine positive influences (e.g., social support, resilience) to test vantage sensitivity models and offer a more comprehensive understanding of adaptation among asylum seekers.

Despite these limitations, our study offers important contributions to the literature on asylum seekers by highlighting environmental sensitivity as a key individual difference shaping these underrepresented individuals' psychological responses to postmigration stressors and intolerance of uncertainty, two defining features of their existential condition.

## 7 Implications

From an applied perspective, the findings confirm that material and interpersonal stressors experienced in the post-settlement situation, in addition to pre-migration traumatic events, can negatively affect asylum seekers' psychosocial adjustment, as evidenced also by prior research (Gleeson et al., 2020). Of note, some of the stressors most frequently experienced in our sample (i.e., being unable to find a job, family separation and concerns, loneliness) were found to have strong and consistent associations with unfavorable mental health outcomes in a recent systematic review conducted in the European context (Nowak et al., 2023). Given that factors such as access to the labor market and family reunification are subject to immigration and reception policies, it is paramount for local institutions to reduce barriers and inequalities that impede the effective inclusion of these individuals into the host society. Furthermore, the fact that more than half of the participants described the delays in their asylum application as highly stressful calls for the need to strengthen mental health support among asylum seekers, who have been found to show a greater prevalence of mental health issues compared not only to the general population, but also to the refugee population (i.e., individuals who were granted refugee status; Turrini et al., 2017).

In terms of clinical implications, while PTSD treatment in emergency settings has often focused on avoidance symptoms, the relation between post-migration factors and negative cognitions/affect, intrusion, and hyperarousal (but not avoidance) found in our study suggests that tailored interventions to reduce these cluster symptoms might be more relevant and effective for this specific population, i.e., asylum seekers who have spent a medium to long period of time in the resettlement country (see Specker et al., 2024). The fact that our models accounted for a meaningful proportion of variance in these symptom clusters underscores the practical relevance of addressing these issues in clinical and preventive contexts. Moreover, the detrimental role of intolerance of uncertainty in both negative cognitions/affect and hyperarousal supports the idea that the (in)ability to deal with uncertain situations is essential in the wellbeing of immigrant youth seeking international protection, whose condition is intrinsically characterized by uncertainty surrounding their legal status (Ceccon and Moscardino, 2024). Hence, the implementation of intervention programs aiming to boost asylum seekers' coping strategies in the face of uncertainty might protect them from the adverse effects of excessive worry (see Wahlund et al., 2020). Such interventions could be particularly beneficial for highly sensitive individuals who, according to our findings, are more prone to experience intense negative emotions and cognitions, but have also been shown to respond more positively to psychosocial and preventive programs targeting internalizing symptoms and emotion regulation strategies (Kibe et al., 2020; Pluess and Boniwell, 2015). Building on the principle of vantage sensitivity, our findings support the development of tailored interventions that could enhance support for asylum seekers based on their sensitivity levels. Highly sensitive individuals, given their heightened reactivity, may benefit most from emotion regulation training, supportive counseling, and efforts to reduce uncertainty through clear communication and structured routines. In contrast, less sensitive individuals might respond well to psychoeducation and resilience-focused workshops.

## 8 Conclusion

This study suggests that postmigration stressors and intolerance of uncertainty affect PTSD symptom clusters in distinct ways, depending on asylum seekers' level of environmental sensitivity. Specifically, while low sensitive individuals exhibited fewer avoidance symptoms in the face of stress, high sensitivity amplified the negative impact of uncertainty on affective and cognitive symptoms. These results underscore the need to address modifiable postmigration stressors, such as prolonged waiting times and restricted work permissions. Possible policy and practice recommendations include streamlining asylum application procedures, providing clear and consistent information about legal status and rights, and integrating basic psychological screening into routine health or intake assessments to identify individuals at greater risk. Reception facilities could also implement low-cost, sensitivity-informed supports (e.g., structured routines, designated quiet spaces, or peer-led groups) that help buffer stress among highly sensitive individuals. In line with the peak-end rule (Kahneman et al., 1993), which suggests that people's retrospective evaluations of stressful experiences are shaped by their most intense and final moments, reducing peak adversity (e.g., detention or extreme uncertainty) and improving the resolution phase (e.g., faster, more transparent asylum decisions) may help lessen lasting psychological distress. Taken together, the findings highlight the potential of scalable, targeted strategies to improve mental health outcomes and reduce the systemic burden of prolonged uncertainty on both individuals and reception systems.

## Data Availability

The raw data supporting the conclusions of this article will be made available by the authors, without undue reservation.
